# A Network-Based Method for Mechanistic Investigation and Neuroprotective Effect on Post-treatment of Senkyunolid-H Against Cerebral Ischemic Stroke in Mouse

**DOI:** 10.3389/fneur.2019.01299

**Published:** 2019-12-19

**Authors:** Jie Zhang, Yunyao Jiang, Nan Liu, Ting Shen, Hyo Won Jung, Jianxun Liu, Bing Chun Yan

**Affiliations:** ^1^Jiangsu Key Laboratory of Integrated Traditional Chinese and Western Medicine for Prevention and Treatment of Senile Diseases, Department of Traditional Chinese and Western Medicine, Yangzhou University, Yangzhou, China; ^2^School of Pharmaceutical Sciences, Institute for Chinese Materia Medica, Tsinghua University, Beijing, China; ^3^Beijing Increase Research for Drug Efficacy and Safety Co., Ltd., Beijing, China; ^4^School of Life Sciences, Huaiyin Normal University, Huai'an, China; ^5^Department of Herbology, College of Korean Medicine, Dongguk University, Gyeongju-si, South Korea; ^6^Korean Medicine R&D Center, Dongguk University, Gyeongju-si, South Korea; ^7^Beijing Key Laboratory of TCM Pharmacology, Xiyuan Hospital, China Academy of Chinese Medical Sciences, Beijing, China; ^8^Department of Neurology, Affiliated Hospital, Yangzhou University, Yangzhou, China; ^9^Jiangsu Key Laboratory of Zoonosis, Jiangsu Co-innovation Center for Prevention and Control of Important Animal Infectious Diseases and Zoonoses, Yangzhou, China

**Keywords:** senkyunolide-H, cerebral ischemic stroke, network pharmacology, PI3K/Akt/NF-κB pathway, neuroprotection

## Abstract

Senkyunolide-H (SEH), a major bioactive compound extracted from *Ligusticum* chuanxiong, has been reported to be effective in preventing cerebral ischemic stroke (CIS). In this study, we employed network pharmacology to reveal potential mechanism of SEH against CIS on a system level and confirmed the therapeutic effects of SEH on CIS by models of cerebral ischemia-reperfusion *in vivo* and *in vitro*. Through protein-protein interaction networks construction of SEH- and CIS-related targets, a total of 62 key targets were obtained by screening topological indices and analyzed for Gene Ontology and Kyoto Encyclopedia of Genes and Genomes pathway enrichment. Gene Ontology analysis indicated that SEH might have a role in treating CIS via regulating some biological processes including regulation of transcription from RNA polymerase II promoter, epidermal growth factor receptor signaling pathway, phosphatidylinositol-mediated signaling, and some molecular function, such as transcription factor and protein phosphatase binding and nitric oxide synthase regulator activity. Meanwhile, the Kyoto Encyclopedia of Genes and Genomes analysis showed that phosphoinositide 3-kinase (PI3K)/protein kinase B (Akt) signaling pathway was significantly enriched. In addition, our result showed that SEH posttreatment significantly decreased the neurological scores, infarct volume, and neuronal death in the middle cerebral artery occlusion mice. Moreover, the PI3K/Akt/nuclear factor kappa B signaling pathway was activated by intragastric administration of 40 mg/kg SEH, as verified by Western blot. *In vitro*, treatment of PC12 cells with 100 μM SEH markedly reduced cell death induced by oxygen-glucose deprivation through the activation of PI3K/Akt/nuclear factor kappa B pathway, and the therapeutic effect of SEH was obviously inhibited by 10 μM LY294002. In summary, these results suggested that SEH carries a therapeutic potential in CIS involving multiple targets and pathways, and the most crucial mechanism might be through the activation of PI3K/Akt/nuclear factor kappa B (NF-κB) signaling pathway to inhibit inflammatory factor releases and increase the antiapoptosis capacity. Our study furnishes the future traditional Chinese medicine research with a network pharmacology framework.

## Introduction

Stroke is reaching epidemic proportions in the world, marked by high incidence, death, and disability rates (1). Responsible for ~88% of global stroke subtypes, cerebral ischemic stroke (CIS) occurs in a cerebral vascular obstruction, which causes pathological processes such as metabolic disorders, inflammation, and cellular apoptosis, and leads to severe neurological symptoms (2). Rapid restoration of the cerebral blood flow and neuronal protection are the major therapeutic strategies for CIS (3). Until now, recombinant tissue plasminogen activator is the only thrombolytic drug approved by the US Food and Drug Administration for CIS treatment (4). However, simply 5% of patients benefit from recombinant tissue plasminogen activator therapy owing to the time window limitations (<4.5 h) and fatal side effects of reperfusion injury (5). Because of the huge burden of social and economic diseases, traditional Chinese medicine (TCM) serves as an important replacement or complementary therapy in many countries. As multicomponent and multitarget medicine, TCM basically achieves the therapeutic effect via conjointly regulating the molecular network of body system with its active ingredients (6). In recent years, greater attention has been paid to monomer pharmacological effects as the development of extract technology.

**Figure 1:**
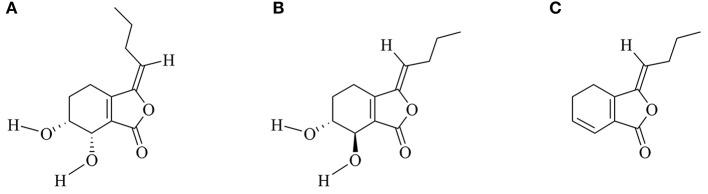
Chemical structures of SEH **(A)**, SEI **(B)**, and LIG **(C)**. SEH, senkyunolide-H; SEI, senkyunolide-I; LIG, Z-ligustilide.

Senkyunolide-H (SEH, Figure 1A) is one of the major bioactive components of TCM Ligusticum chuanxiong Hort, which is widely used to treat migraine, anemia, and cardiovascular and cerebrovascular diseases in China (7, 8). It can enter the blood and cerebrospinal fluid quickly with its well fat and water solubility. Recent experimental studies have shown that SEH could alleviate neuroinflammatory injury induced by intracerebral hemorrhage and activate endogenous cytoprotective mechanism against oxidative damage in human liver HepG2 cells (8, 9). Intriguingly, SEH and its stereoisomer senkyunolide-I (Figure 1B) are primary metabolite of Z-ligustilide (Figure 1C), which holds antiapoptotic ameliorative effects on focal cerebral ischemia *in vitro* and *in vivo* via activating the phosphoinositide 3-kinase (PI3K)/protein kinase B (Akt) and mitogen-activated protein kinase (MAPK) pathways (10). Furthermore, researches by Yangye Hu suggested that senkyunolide-I exhibited definite antiapoptotic biological activity on cerebral ischemia/reperfusion injury *in vivo* and anti-inflammatory effects against endotoxin insult *in vitro* (11, 12). Despite its higher stability and bioavailability compared with Z-ligustilide (9, 13), no published information to date has been carried out to explore the possible mechanisms of SEH on CIS.

**Figure 2:**
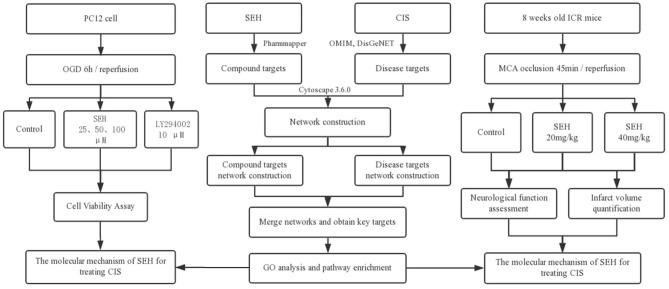
The flowchart of this study based on an integration strategy of network pharmacology and experimental verification for deciphering pharmacological mechanisms of SEH acting on CIS. SEH, senkyunolide-H; CIS, cerebral ischemic stroke; OMIM, Online Mendelian Inheritance in Man; DisGeNET, a database of gene-disease associations; OGD, oxygen-glucose deprivation; MCA, middle cerebral artery.

Network pharmacology is emerging as a prospective strategy, one that combines systematic methods for the exploration of multichannel signaling pathways regulation (14, 15). Achieving an agent targets network from an overall and comprehensive angle is its greatest advantage (16). In addition, it helps to understand the polypharmacology of a drug, increase drug curative effect and success rate of clinical tests, and reduce the discovery costs. Until now, lots of researches on exploring the molecular mechanisms of TCM formulae and investigating effective components from traditional herbs have been published, such as Tian-Ma-Gou-Teng-Yin against Alzheimer's disease (17), Tong Sheng tablets against cerebral ischemia reperfusion injury (18), and quercetin for cardiovascular disease treatment (19). Therefore, we are going to investigate the mechanisms of SEH treatment on CIS with the novel network pharmacology program, including herbal target prediction, disease target collection, protein-protein interaction (PPI) network construction, topological feature analysis, and key target functional characterization, and then to validate its therapeutic effect on *in vivo* and *in vitro* models of CIS (Figure 2).

## Materials and Methods

### Predicting SEH Potential Targets

The structure of SEH (PubChem CID: 10036567) was downloaded from the NCBI PubChem database (https://pubchem.ncbi.nlm.nih.gov/) and input onto the PharmMapper server (http://lilab.ecust.edu.cn/pharmmapper/) in a MDL sdf. format file, which was designed for identifying possible targets of small molecules via a reverse pharmacophore matching approach (20). The species was limited to “*Homo sapiens*,” and the first 100 potential targets were ranked according to the fitted scores for subsequent research. Owing to the non-standard naming, we got corresponding official symbols through UniProtKB database (http://www.uniprot.org/), which is the central hub for collecting protein function information with exact, consistent, and abundant annotation (21).

### Mining Known CIS-Associated Targets

CIS-related known targets were mined from the two available databases, OMIM database (Online Mendelian Inheritance in Man, http://www.omim.org) and DisGeNET database (http://www.disgenet.org/). We used “cerebral infarction” and “cerebral ischemic stroke” as the keywords, respectively, for genes searching, respectively (22, 23).

### Constructing PPI Networks and Analyzing Network Topological Features

To provide the scientific and reasonable interpretation of the complex relationships between the targets of SEH and CIS, networks construction and analysis were carried out as previously described (24). PPI networks of SEH targets and CIS targets were constructed by Cytoscape3.6.0 software (National Institute of General Medical Sciences, United States) new plugin BisoGenet, which integrates six kinds of PPI databases: the IntAct Molecular Interaction Database (IntAct), Molecular Interaction Database (MINT), the Biological General Repository for the Database of Interacting Proteins (DIP), the Biomolecular Interaction Network Database (BIND), the Human Protein Reference Database (HPRD), and Interaction Datasets (BioGRID). Then, the two networks were merged to acquire important targets. After the network analysis, we chose the nodes the degree of which is more than two-fold the median degree of all the nodes as big hubs. Subsequently, the topological features were calculated by degree centrality (DC), betweenness centrality (BC), and closeness centrality (CC) (DC values are twice larger than the median value, BC and CC value larger than the median value of all the network nodes) to screen the putative targets for key targets.

### Gene Ontology and Pathway Enrichment Analysis

The Database for Annotation, Visualization and Integrated Discovery (DAVID) (https://david.ncifcrf.gov/) were applied for Gene Ontology (GO) enrichment and Kyoto Encyclopedia of Genes and Genomes (KEGG) pathway analysis of key targets. This involves a two-step operation: first, the ID of gene was inputted and the species was specified as “*Homo sapiens*;” second, enrichment analysis was executed using functional annotation tool. Three GO enrichment categories of biological process, molecular function, and cellular component were selected to draw bar charts. A bubble chart of top 20 pathways was made by OmicShare platform (http://www.omicshare.com/).

### Experimental Animals

Comparative Medicine Center of Yangzhou University (Yangzhou, China) supplied 8-week-old male Institute of Cancer Research mice (25-30 g), and all mice were domesticated for 7 days at least. The environment was maintained at room temperature of 23°C, humidity of 60%, and adequate free forage and water. To simulate a normal growing environment, we made a light-dark cycle that changes every 12 h. All experimental procedures were carried out in accordance with the guidelines of the National Institutes of Health Guide for the Care and Use of Laboratory Animals and were approved by the Yangzhou University-Institutional Animal Care and Use Committee (YIACUC-14-0015) based on ethical procedures and scientific nursing.

### Transient Cerebral Ischemia Model

Isoflurane (3-4%) (RWD Life Science, Guangdong Province, China) in 30% oxygen and 70% nitrous oxide was used to anesthetize the animal. Then, the right common carotid artery, vagus nerve, external carotid artery, and internal carotid artery were exposed and separated carefully after a median incision of the neck skin. A monofilament nylon filament was inserted into the internal carotid artery through an incision of external carotid artery until the middle cerebral artery (MCA) occluded and taken out to commence reperfusion after 45 min infraction. Sham-operated groups were given the same surgery without inserting the filament. The whole surgery would maintain the body temperature of mice at 37.0-37.5°C by a rectal thermometer connected to an automated heating pad [i.e., transient MCA occlusion (MCAO)/reperfusion was induced by a filament insertion/removal in the carotid artery]. Thereafter, the mice were incubated in the thermal incubator to maintain the body temperature up to euthanasia.

### Groups and Drug Administration

According to previous studies (11, 25, 26), mice were randomly divided into the following groups (*n* = 14 in each group): sham operation (sham group); sham operation with 40 mg/kg SEH treatment (sham-SEH group); MCAO treatment (MCAO group); MCAO with 20 mg/kg SEH treatment (20 SEH group); and MCAO with 40 mg/kg SEH treatment (40 SEH group). SEH was administered intragastrically at the start and end of ischemic surgery immediately. Equal volume of 0.9% saline was intragastrically injected into two groups of mice (sham and MCAO). Mice were killed at 6 and 24 h after reperfusion. SEH (Shanghai Standard technology Co., Ltd, Shanghai, China) was dissolved in 0.9% saline and 0.1% dimethyl sulfoxide, and the purity was above 98.32%.

### Neurological Score

The neurological function of mice was assessed in accordance with the Bederson scale before killing. Four levels were divided in Bederson scale (27): level 0, no significant changes; grade 1, abnormal bending of the forelimbs; level 2, poor resistance to lateral thrust, but no circling; and level 3, poor resistance to lateral thrust with circling. Blinded assessment was conducted at a specified time. The animal model is built successfully if the scores are greater than or equal to level 1.

### TTC Staining and Quantifying Infarct Volume

The procedure is following that described above (28). The mice were killed with 10% chloral hydrate (Aladdin, Shanghai, China), and decapitation was performed immediately after full reperfusion (1 day). We took the brain very carefully, and then, the brain was weighed and cut into 2-mm thick slices. Two percent 2,3,5-triphenyl tetrazolium chloride (Sigma-Aldrich, St. Louis, MO) was used for staining for half an hour under dark condition. After staining, we transferred the brain slices into 4% formalin overnight. The availability of the slice was determined by the slice color; red color of the brain slice was available, while white color of the brain slice was inactivated. Image J software was used for measuring the infarct volume and whole area volume of brain slice. The infarct volume was calculated by multiplying the increased infarct size per slice by slice thickness (2 mm). The results were shown as (infarct volume/whole brain volume) × 100%.

### Tissue Processing for Histology

We collected the tissues according to the method described in the previous study (29), and 10% chloral hydrate (Aladdin, China) was used to maintain anesthesia in the mice; subsequently, 0.1 M phosphate-buffered saline (PBS, pH 7.4) and 4% paraformaldehyde in 0.1 M phosphate buffer (pH 7.4) were perfused transcardially. The brains of mice were removed in the same fixative solution and fixed for 4 h and cryoprotected by 30% sucrose infiltration overnight. Thereafter, frozen tissues were continuously sliced into 30-μm coronal sections via a cryostat (Leica, Wetzlar, Germany) and then collected into six porous plates containing PBS.

### NeuN Immunohistochemistry

Immunohistochemistry was conducted according to our formerly published program (29). The slices were first treated with 0.3% hydrogen peroxide (H_2_O_2_) diluted in PBS for 20 min and 5% normal serum diluted in 0.01 M PBS for 30 min. Then, the slices were incubated successively with diluted rabbit antineuronal nuclei (anti-NeuN) (1:1,000, Cell Signaling Technology) overnight at 4°C, biotinylated goat antirabbit IgG (1:250, Vector, Burlingame, CA), and streptavidin peroxidase complex (1:200, Vector). After that, the tissues were dyed with 3,3′-diaminobenzidine tetrahydrochloride in 0.01 M PBS and dehydrated on the adhesion microscope slides. Finally, we used neutral gum (Solarbio, Beijing, China) sealing piece. Digital images of CA1 region were observed with a computer-based microscope (Nikon, Chiyoda-Ku, Tokyo, Japan), which is equipped with an image-analyzing system. Cell counts were gained through averaging the counts from the sections of each animal. The staining intensity of NeuN immunoreactive structures was assessed on the basis of an optical density (OD), which was obtained after the transformation of the mean gray level using the formula: OD = log (256/mean gray level). The OD of the background was taken from areas near the measured area. After the background density was subtracted, a ratio of the OD of image file was calibrated as percent [relative OD (ROD)] through Adobe Photoshop version 8.0 and then analyzed by NIH Image 1.59 software. We normalized each sample against the level of vehicle-sham sample. All measurements were performed under the same conditions by two observers in blind conditions to ensure objectivity.

### PC12 Cell Culture and OGD Model

PC12 cell line (rat pheochromocytoma) was purchased from the American Type Culture Collection, seeded at 1 × 10^6^ cells/well in six-well plates and cultured at 37°C in a damp atmosphere containing 5% CO_2_. Cells were grown in Dulbecco's modified Eagle's medium (DMEM) (Gibco, Grand Island, NY) included penicillin (100 U/ml), 5% heat-inactivated fetal bovine serum (Gibco), streptomycin (100 mg/ml), and 10% horse serum (Gibco). To simulate the model of cerebral ischemia *in vitro*, cells were incubated in the serum/glucose-free DMEM after being seeded overnight and washed twice with PBS (pH 7.2) and then transferred to a hypoxic chamber (Thermo Fisher, Waltham, MA) containing 95% N_2_ and 5% CO_2_ for 6 h. After oxygen-glucose deprivation (OGD) exposure, the cells were incubated with conditioned DMEM at 37°C in a damp atmosphere containing 5% CO_2_ for 24 h reperfusion. In addition, the cells were divided into different groups: the sham group, the OGD group, the OGD + SEH groups (25, 50, and 100 μM, respectively), and the OGD + SEH + LY294002 (10 μM) groups. The different concentrations of SEH and LY294002 (MCE MedChem Express, Monmouth, NJ) were added throughout the OGD-reperfusion (OGD/R) treatment within the standard medium. The vehicle group was subjected to the same experimental procedures without exposure to the serum/glucose-free DMEM medium and any drugs. Then, the cells were collected for Western blotting detection.

### CCK8 Cell Viability Assay

Cell Counting Kit-8 was used for measuring cell viability (CCK8; Dojindo Laboratories, Kumamoto, Japan). Cells were seeded into a 96-well plate at a density of 6 × 10^3^ cells/100 μl. After stabilizing, cells were exposed to OGD/R in the absence or presence of indicated concentrations of SEH and LY294002 treatment. After 24-h reperfusion, 10 μl of CCK8 solution was included to each hole (100 μl medium), incubated at 37°C for 2 h, and then, the absorbance was measured at 450 nm in a multimode plate reader (Espier, PerkinElmer, Singapore).

### Western Blot Analysis

Western blot experiment was carried out on the basis of a procedure published before (28). The mice brains (*n* = 7 in each group) were cut into 400-μm thickness serially and transversely on a vibratome, and the hippocampus was carved with a scalpel. We preprocessed the tissues and the whole cell lysates from PC12 cell by Whole Cell Lysis Assay kit (KeyGEN, Nanjing, China)/Total Protein Extraction Kit and measured the protein concentration with a Pierce BCA Protein Assay Kit (Thermo Scientific, USA). The same amount of protein (40 μg) was separated by suitable percentage sodium dodecyl sulfate polyacrylamide gel electrophoresis and transferred to nitrocellulose membranes (Millipore, Bedford, USA). For a clear band, the membranes were cultured in Tris-buffered saline containing 5% bovine serum albumin and 0.1% Tween 20 for 60 min, then incubated in primary antibody overnight at 4°C, and followed in the corresponding secondary antibody at room temperature for 2 h. Protein expression was detected by SuperSignal West Pico chemiluminescent substrate (Thermo Scientific, Rockford, USA).

Densitometric analysis on all scanned Western blot results was performed using Quantity One Analysis Software (Bio-Rad). Relative OD (ROD) calculations are mainly followed: the ratio of the calibrated ROD is expressed in percentage, and the sham group is defined as 100%. Three similar independent experiments were represented by each blot results at least. The primary and secondary antibodies were listed as follows: rabbit anti-PI3K (1:1,000, Cell Signaling Technology) and p-PI3K (1:1,000, Cell Signaling Technology), rabbit anti-Akt (1:1,000, Cell Signaling Technology) and p-akt (1:1,000, Cell Signaling Technology), rabbit anti-NF-κB (1:1,000, Abcam), rabbit anti-Bcl-xL (1:1,000, Cell Signaling Technology), rabbit anticleaved caspase 3 (1:1,000, Cell Signaling Technology), anti-β-actin (1:3,000, Abcam), and goat antirabbit IgG (Santa Cruz, USA).

### Statistical Analysis

The data shown in the study represent the means ± SD and were plotted in histograms with GraphPad Prism 7.0. Differences in average values among groups were analyzed through one-way analysis of variance test by SPSS software. *p* < 0.05 was considered to have statistical significance.

## Results

### SEH Potential Targets Prediction and CIS-Associated Targets Collection

A total of 11 potential targets were derived and screened with a fit score value >4.5 from the top 100 potential human protein targets of SEH through PharmMapper server. Detailed information is described in Supplementary Table 1. By means of OMIM and DisGeNET databases, we obtained 11 and 130 ischemic-stroke-related targets, respectively. A detailed information after filtered overlapping protein targets from the above two available resources is described in Supplementary Table 2.

### PPI Networks Construction and Network Topological Parameters Analysis

**Figure 3:**
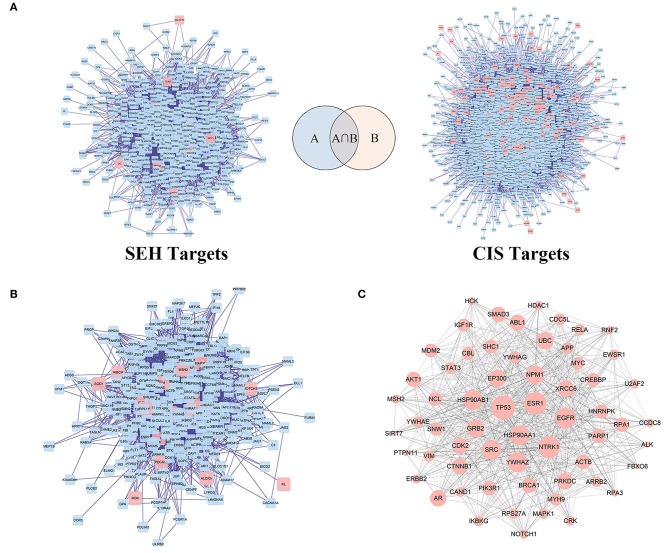
The PPI networks construction and analysis for SEH against CIS. **(A)** PPI networks of SEH-related targets and CIS targets. **(B)** The interactive PPI network of SEH and CIS targets gained from **(A)**. **(C)** The PPI network of key targets of SEH acting on CIS obtained from **(B)**. Pink square node in **(A,B)** represent direct targets of SEH and CIS. Blue nodes represent indirect targets of SEH and CIS. Pink circular nodes in **(C)** represent key targets of SEH acting on CIS, while its size is proportional to the significance. Edges represent interactions among SEH and CIS targets. PPI, protein-protein interaction; SEH, senkyunolide-H; CIS, cerebral ischemic stroke.

As the charts show (Figure 3A, Supplementary Tables 3, 4), SEH targets PPI network, and CIS-related target PPI network was constructed by BisoGenet to find out the genes with direct or indirect effects. There were 639 nodes and 7,724 edges in the SEH targets network. Meanwhile, network of CIS-related targets had 3,605 nodes and 89,245 edges. To reveal the relationship between SEH and CIS, a new network (Figure 3B) was merged from the two networks (Figure 3A) with the merge function of Cytoscape3.6.0, which included 352 overlapping targets and 4,754 edges. The common targets were not only the important goal of SEH treatment in CIS but also the vital targets screened for further research. Subsequently, the topological feature values of common targets in the network (Figure 3B, Supplementary Table 5) including DC, BC, and CC were analyzed for the important key protein targets. Finally, a total of 62 nodes, which DC > 44, BC > 0.001, and CC > 0.470, were chosen as the key targets (Figure 3C, Supplementary Table 5).

### GO Enrichment Analysis of Candidate Targets

**Figure 4:**
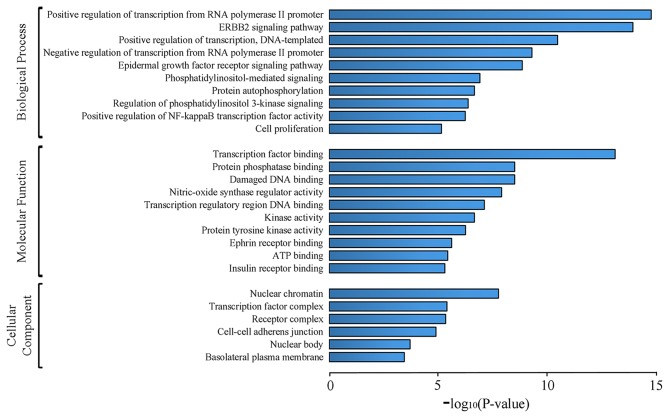
GO analysis of candidate targets. The top 10 terms for biological processes, molecular function, and top 6 terms for cell component with *p* < 0.001 are shown. *X*-axis indicates *p*-values of GO enrichment for each subcategory.

GO analysis of 62 candidate targets for SEH treating on CIS was performed via the DAVID database to investigate their functions and inherent significance in the biological system networks. The diagram (Figure 4, Supplementary Table 6) contained three parts, which were biological process, cellular component, and molecular function. We found that biological processes were related to the regulations of RNA polymerase II promoter transcription, DNA-templated transcription, NF-κB transcription factor activity, protein autophosphorylation, and signaling pathways of PI3K, ErbB2, epidermal growth factor receptor, and phosphatidylinositol mediated. The molecular function was related to the bindings of transcription factor, damaged DNA, protein phosphatase, transcription regulatory region DNA, ephrin receptor, ATP and insulin receptor, and activities of nitric oxide synthase regulator, kinase, protein tyrosine kinase, and cell proliferation. Finally, the cellular component was related to nuclear chromatin, transcription factor complex, receptor complex, cell-cell adherens junction, nuclear body, and basolateral plasma membrane.

### KEGG Pathway Enrichment Analysis for Key Targets

**Figure 5:**
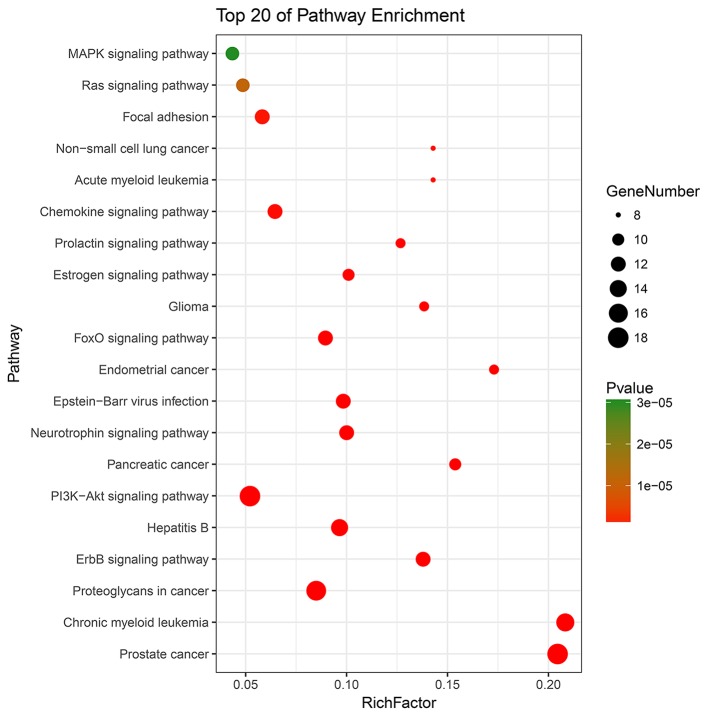
The top 20 terms for KEGG signaling pathway enrichment of major targets. “Rich factor” indicates the ratio of the number of target genes belonging to a pathway and the number of the annotated genes located in the pathway. A higher rich factor reflects a higher level of enrichment. The size of the dot is proportional to the number of key targets in the pathway, and the color of the dot refers to the *p*-value.

All 62 key targets were significantly enriched onto 49 pathways with the adjusted *p* < 0.01 by means of DAVID database (Supplementary Table 7). Top 20 KEGG pathways were picked and constructed in bubble diagram on the basis of *p*-value and number of key targets included. The picture (Figure 5) indicated the mechanisms that might be concerned with PI3K/Akt, ErbB, neurotrophin, FOXO, and estrogen signaling pathways. Detailed information was described in Supplementary Table 7.

### The Neuroprotective Effects of SEH on CIS Mice Model

**Figure 6:**
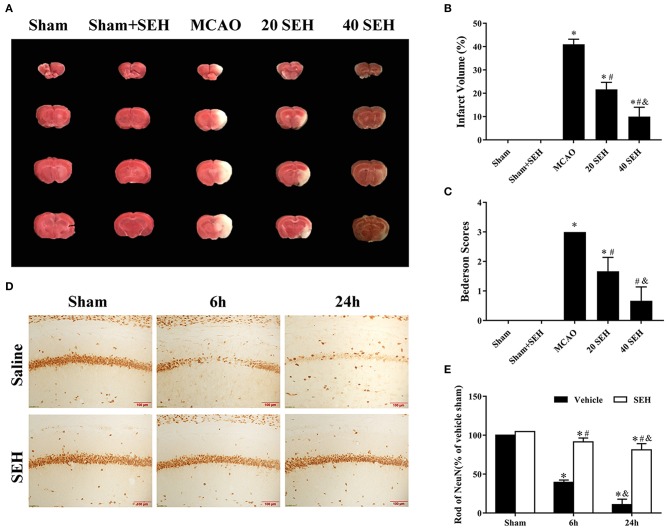
The neuroprotective effects of SEH. **(A)** 2,3,5-Triphenyl tetrazolium chloride (TTC) staining in the sham, sham + SEH, MCAO, 20 and 40 mg/kg (intragastric administered) SEH-MCAO groups. **(B)** The infarct volume was expressed as the ratio of (infarct volume/the whole brain volume) × 100% (*n* = 7 per group; *p < 0.05: significantly different from the sham group; ^#^*p* < 0.05: significantly different from the MCAO group). Bars indicate mean ± SD. **(C)** Bederson neurological score in the sham, MCAO, and 20 and 40 mg/kg (intragastric administered) SEH-MCAO groups (*n* = 7 per group; *p < 0.05: significantly different from the sham group; ^#^*p* < 0.05: significantly different from the MCAO group). Bars indicate mean ± SD. **(D)** Immunohistochemistry for NeuN in the hippocampus CA1 region of the sham, MCAO, 40 mg/kg intragastric administered SEH-sham, and 40 mg/kg intragastric administered SEH-MCAO groups. **(E)** The ratio of NeuN immunoreactivity. Scale bar = 100 μm (*n* = 7 per group; *p < 0.05: significantly different from the corresponding sham groups; ^#^*p* < 0.05: significantly different from the corresponding vehicle-MCAO groups; ^&^*p* < 0.05: significantly different from the corresponding 6 h-MCAO groups). Bars indicate mean ± SD.

The staining 2,3,5-triphenyl tetrazolium chloride was frequently used for the evaluation of infarct volume after brain ischemia. As shown in Figures 6A,B, in the 20 mg/kg SEH treatment ischemia group, the sizes of infarct regions were ~23% of whole brain volume and markedly lower than those of MCAO group (accounting for 43% of whole brain volume). Meanwhile, only few infarct sizes (~9% of the brain volume) were observed in the treatment of 40 mg/kg SEH ischemia group. Besides, we evaluated the neurological function of mice according to the Bederson scale (0-3) before killing. We observed that mice treated with 40 mg/kg SEH obtained better scores (the score of neurological function was 1), which was much lower than that in the MCAO group (the score of neurological function was 3) (Figure 6C). In addition, neuronal death in the hippocampus was observed by immunohistochemistry of NeuN. Most immunoreactions of NeuN cells were lost in the CA1 region of hippocampal in the reperfusion at 6 and 24 h after MCAO; however, in the 40 mg/kg SEH-MCAO group, many survival neurons were found in the CA1 region of hippocampal compared with that in the MCAO group (Figures 6D,E). These results indicated that SEH treatment reduced the infarct volume and improved neurological deficits after cerebral IR in mice, and the neuroprotection of SEH was especially embodied in the 40 mg/kg SEH-MCAO group.

### Role of PI3K/Akt/NF-κB Signaling-Related Protein Levels in MCAO Mice After SEH Treatment

**Figure 7:**
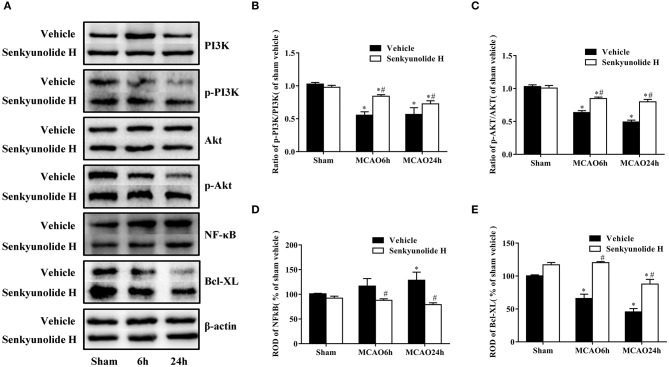
Effect of SEH treatment on changes of PI3K/Akt/NF-κB signaling pathway and antiapoptotic levels in the hippocampus region. **(A)** Protein bands of PI3K, p-PI3K, Akt, p-Akt, NF-κB, and Bcl-XL in each group. **(B)** The ratio of p-PI3K/PI3K. **(C)** The ratio of p-Akt/Akt. **(D)** NF-κB expression. **(E)** Bcl-XL expression. Relative optical density (ROD) as a percentage of the immunoblot band is presented (*n* = 7 per group; *p < 0.05: significantly different from the corresponding sham group; ^#^*p* < 0.05: significantly different from the corresponding vehicle-MCAO group). Bars indicate mean ± SD.

To verify the mechanism by which SEH improved nerve damage after ischemic brain injury, Western blot analysis was used to measure relative protein expression levels. As shown in Figure 7, the PI3K expression was obviously decreased, and the p-PI3K level increased in the hippocampus region of the SEH treatment groups than that of the MCAO groups. Variation in the p-Akt/Akt protein ratio was consistent with the change in the p-PI3K/PI3K protein ratio. Yet, the SEH treatment groups exhibited markedly reduced expression of NF-κB while enhanced expression of Bcl-XL. The above data suggested that SEH treatment protected against IR injury was related to the PI3K/Akt/NF-κB signaling pathway activation.

### Cell Viability and the Protein Levels of Cleaved Caspase3, Bcl-XL, PI3K/Akt/NF-κB Signaling of OGD/R-Injured PC12 Cells After SEH and LY294002 Treatment

**Figure 8:**
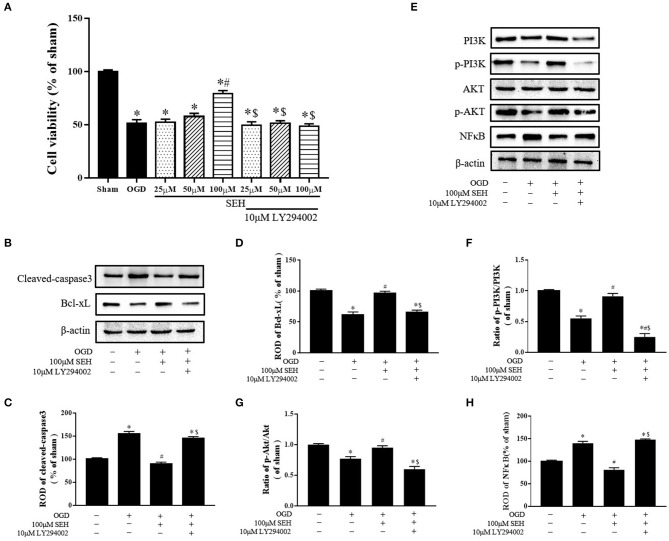
Effect of SEH and LY294002 treatment on cell viability, apoptotic levels, and PI3K/Akt/NF-κB signaling pathway protein of OGD/R-injured PC12 cells. **(A)** The cell viability was measured by CCK-8 assay in the PC12 cells after OGD/R. **(B)** Protein bands of cleaved caspase 3 and Bcl-XL in each group. **(C)** Cleaved caspase 3 expression. **(D)** Bcl-XL expression. **(E)** Protein bands of PI3K, p-PI3K, Akt, p-Akt, and NF-κB in each group. **(F)** The ratio of p-PI3K/PI3K. **(G)** The ratio of p-Akt/Akt. **(H)** NF-κB expression. Relative optical density (ROD) as a percentage of the immunoblot band is presented (*n* = 7 per group; *p < 0.05: significantly different from the sham group; ^#^*p* < 0.05: significantly different from the OGD group; ^$^*p* < 0.05: significantly different from the corresponding SEH treatment group). Bars indicate mean ± SD.

The possible neuroprotective mechanisms of SEH on OGD/R injured PC12 cells were determined via the CCK8 assay and the Western blot analyses of apoptosis-related proteins and PI3K/Akt/NF-κB signaling expressions. As shown in Figure 8A, cell viability was obviously decreased in the OGD group compared with the sham group but gradually increased in the SEH-treated OGD/R groups with dose-dependent SEH concentration. In the groups of different SEH concentrations with 10 μM LY294002, the cell viability was significantly reduced compared with the only SEH treatment group. In addition, the levels of cleaved caspase 3 were markedly upregulated by OGD/R and downregulated by OGD/R with 100 μM SEH treatment. However, in the group including 10 μM LY294002 treatment, the level of cleaved caspase 3 was similar to that of only OGD/R treatment group (Figures 8B,C). Furthermore, we observed that the Bcl-XL expressions were opposite to the cleaved caspase 3 expressions in the PC12 cells under OGD/R. Besides, the ratios of p-PI3K/PI3K and p-Akt/Akt, as well as the ROD of Bcl-XL, were significantly increased in the group treated with 100 μM SEH compared with the OGD group. However, these increases were inhibited by 10 μM LY294002 (Figures 8D–G). Moreover, the level of NF-κB was significantly upregulated by OGD/R treatment, which was blunted by 100 μM SEH treatment. After the treatment with 10 μM LY294002, the level of NF-κB was increased compared with the only SEH treatment group (Figure 8H). Together with the abovementioned data, these results suggested that a dose of 100 μM SEH is sufficient to markedly decrease OGD/R injury and that the mechanism of SEH neuroprotective effect on OGD/R-injured PC12 cells is related to the activation of the PI3K/Akt/NF-κB pathway and inhibited by LY294002.

## Discussion

Ischemic stroke is a main cause of death and disability and a common central nervous system disease with poor prognosis. SEH is a bioactive compound extracted from the TCM *Ligusticum* chuanxiong Hort. In clinic, the treated duration of *Ligusticum* chuanxiong Hort is usually decided by different diseases and the patient's condition. Previous studies reported that 14-60 days for chuanxiong intervention were used for treatment of acute ischemic stroke by hundreds of clinical studies (30). Meanwhile, the meta-analysis suggested that the Ligustrazine (a bioactive component contained in chuanxiong) treatment of 10 days−12 weeks had a significant therapeutic effect on diabetic nephropathy (31). In addition, we found that SEH could be used for many applications. Published researches suggested SEH as a new lead for the development of new antiatherosclerotic drug. For example, SEH showed strong antiproliferative activity in primary culture of mouse aorta smooth muscle cells (32). Recent studies indicated that the absorption of SEH was significantly increased in migrainous rats compared with normal rats (33), and SEH has shown potent neuroprotective effects on intracerebral hemorrhage (9). According to the latest research, SEH could attenuate MPP+-induced neurotoxicity and apoptosis in PC12 cells (34). However, as far as we know, the mechanisms responsible for the SEH function in CIS are not known. To elucidate the mechanism of SEH in CIS, we applied network pharmacology to have a comprehensive and systematic research. A total of 62 key targets of SEH against CIS were obtained and analyzed. GO and KEGG analysis suggested that multiple signaling pathways were involved in the potential mechanisms of SEH. Besides, we confirmed the therapeutic effects of SEH in the mouse MCAO model and PC12 cell OGD/R model.

Through the GO enrichment analysis of the predictive targets of SEH in the treatment of CIS, functions of the key targets and the related pathways information were obtained. It has notable significance for the biological processes such as regulation of transcription from RNA polymerase II promoter and phosphatidylinositol-mediated and epidermal growth factor receptor (EGFR) signaling pathways. Gene transcription regulation is a vital process in CIS. It is coordinated by transcription factors and other proteins and regulates gene functions via a variety of mechanisms, for instance, neurogenesis, inflammation, and angiogenesis (35). Recent study showed that polymorphism (rs145204276) in the promoter of long non-coding RNA growth arrest-specific 5 had a significant association with an increased CIS susceptibility by elevating the transcriptional activity of GAS530197169. EGFR signaling pathway plays critical roles in promoting neurogenesis (36). Autophosphorylation of EGFR activates ERK/MAPK signaling pathway and subsequently leads to cell proliferation and DNA synthesis (37). In Chen's study, astragaloside VI could initiate EGFR/MAPK signaling cascades effectively, accelerate the proliferation of neural stem cells, strengthen motor function, and ameliorate the abilities of learning and memory in rats with transient cerebral ischemic (38). Besides, molecular functions mainly involve transcription factor binding, nitric oxide synthase regulator activity, protein phosphatase binding, and so on. Transcription factor ATF-3 is an appropriate marker for neurons injured by ischemia, and the synergistic effect of ATF-3 and c-Jun may be the key trigger factor for various transcriptional responses to ischemic injury (39). Protein phosphatase binding is closely related to CIS, for example, the insulin-like growth factor 1 receptor often facilitates cell survival, cell proliferation, metabolism, and stress resistance triggered by the tyrosine autophosphorylation of its β chains (40). In addition, our previous findings indicated that insulin-like growth factor 1 receptor may be an alternative target for preventing the cerebral ischemic injury (41).

In KEGG analysis, PI3K/Akt, ErbB, neurotrophin, and FOXO signaling pathways were significantly enriched, and PI3K/Akt signaling pathway may be the most crucial mechanism. The PI3K/Akt pathway is the key to ErbB signal transduction, which controls protein homeostasis through boosting migration, angiogenesis, and cell proliferation. Zhou's data showed that reactive oxygen species eliminated the inhibitory effect of PI3K/Akt signaling pathway on ERK activity during reperfusion, and the strong activation of ERK activity played a pivotal role in cell injury induced by reperfusion (42). In addition, nerve growth factor (NGF) is a member of the neurotrophin family, and NGF/tropomyosin-related kinase A signaling is related to neuronal survival, function, and differentiation (43). Recent study showed that NGF-enhanced angiogenesis contributed to neurological functional recovery after ischemic stroke via the initiation of PI3K/Akt signaling pathway (44). Moreover, PI3K/Akt cascade inhibits the FOXO pathway for regulation of autophagy, metabolism, and oxidative stress. Previous research implicated that FOXO-4 could trigger apoptotic responses or cell cycle arrest via downregulation of Akt, which might be neuroprotective to drive the cells into a state of quiescence during a situation of reduced energy (45).

Lastly, our findings revealed that treatment of MCAO mice with 40 mg/kg SEH, as well as the treatment of OGD PC12 cells with 100 μM SEH, markedly increased p-PI3K, p-Akt, and Bcl-XL protein levels and decreased NF-κB protein expression. Meanwhile, the treatment of OGD/R-injured PC12 cells with 100 μM SEH also reduced cleaved caspase 3 protein expression, and the neuroprotective function of SEH was inhibited by 10 μM LY294002. These data indicated that SEH stimulated PI3K/Akt signaling pathway and inhibited NF-κB signaling pathway in CIS. Currently, it is reported that PI3K/Akt pathway plays a great role in the regulation of cell growth and neurons survival after brain ischemia, demonstrating that pharmacological upregulation of PI3K/Akt signaling could have therapeutic potential for the brain damage (46). Meanwhile, the activation of Akt can promote neuronal proliferation and survival through controlling multiple intracellular signals (47). As a downstream gene of Akt, NF-κB (p65) can be activated by phosphorylated Akt to phosphorylated p65, which causes NF-κB to enter the nucleus, and induces inflammatory response and apoptosis. A previous study has suggested that the NF-κB subunit activation leads to the response of lymphokine-6 and tumor necrosis factor α, which indicated that repressing NF-κB-induced neuroinflammation enhances functional outcomes and alleviates ischemic brain injury (48). Furthermore, recent studies have shown that inhibiting NF-κB could improve the prognosis of stroke, and the inhibitors of NF-κB activation may be possible targets for treatment (49, 50). In addition, Lv's data indicated that CXCL8 gene silencing accelerated the activation of neuroglial cells while suppressing neuroinflammation via the PI3K/Akt/NF-κB pathway in the mice of CIS (50). Besides, LY294002, as a PI3K inhibitor that is widely used for the study of the characteristics of PI3K cell signaling pathways (51–53), inhibited the expressions of PI3K/Akt/NF-κB pathway and blocked the function of SEH treatment on PC12 cells injured by OGD/R. For instance, published study indicated that ginkgolides protected against ischemia-reperfusion damage *in vivo* and *in vitro* through the activation of Nrf2 and CREB via PI3K/Akt signaling, which could be reversed by cotreatment with LY294002 (54). At the same time, LY294002 is also a nonspecific inhibitor that may affect lots of other pathways (55), suggesting that, in addition to the PI3K/Akt pathway, SEH may also activate other pathways to exert therapeutic effects. For example, some studies reported that the neuroprotective effect of SEH was related to the MAPKs pathways (30) and Prx1/TLR4/NF-kB pathway (9). Therefore, our data also verified that SEH treatment could restrain neuronal apoptosis induced by IR through the activation of PI3K/Akt/NF-κB signaling pathway.

To sum up, we explored multiple targets and pathways of SEH treatment against CIS through a network pharmacology approach and confirmed the therapeutic effects of SEH *in vivo* and *in vitro*. Our data indicated that SEH treatment on CIS may be through the activation of PI3K/Akt//NF-κB signaling pathway to inhibit inflammatory factor releases and increase the antiapoptosis capacity. However, owing to the incomplete information in these databases, some targets of SEH or CIS may be ignored and missed during the screening process. In addition, proteins and messenger RNAs validation was not conducted for each of the key targets, and targets that may be of most interest for the SEH treatment of CIS were not identified yet. Hence, future studies will focus more on providing insight into the specific cellular and molecular mechanisms of SEH therapeutic effect on CIS.

## Data Availability Statement

The datasets generated for this study are available on request to the corresponding author.

## Ethics Statement

All experimental procedures were performed according to the guidelines of the National Institutes of Health Guide for the Care and Use of Laboratory Animals and were approved based on ethical procedures and scientific care by the Yangzhou University-Institutional Animal Care and Use Committee (YIACUC-14-0015).

## Author Contributions

JZ and YJ performed the experiments and drafted the manuscript. YJ, NL, TS, HJ, and JL analyzed the data. BY designed the experiments. All the authors discussed the results, reviewed the final manuscript, and approved it for the publication.

### Conflict of Interest

The authors declare that the research was conducted in the absence of any commercial or financial relationships that could be construed as a potential conflict of interest.
